# Evaluation of the efficacy of a commercial inactivated genogroup 2b-based porcine epidemic diarrhea virus (PEDV) vaccine and experimental live genogroup 1b exposure against 2b challenge

**DOI:** 10.1186/s13567-017-0472-z

**Published:** 2017-10-26

**Authors:** Tanja Opriessnig, Priscilla F. Gerber, Huigang Shen, Alessandra Marnie M. G. de Castro, Jianqiang Zhang, Qi Chen, Patrick Halbur

**Affiliations:** 1 The Roslin Institute and The Royal (Dick) School of Veterinary Studies, University of Edinburgh, Midlothian, Scotland, UK; 2 Department of Veterinary Diagnostic and Production Animal Medicine, College of Veterinary Medicine, Iowa State University, Ames, IA USA; 3 Animal Science, School of Environmental and Rural Science, University of New England, Armidale, NSW 2351 Australia; 4 Complexo Educacional Faculdades Metropolitana Unidas, Veterinária, Rua Ministro Nelson Hungria, 541, Real Parque, Morumbi, São Paulo, SP Brazil

## Abstract

Porcine epidemic diarrhea virus strains from the G1b cluster are considered less pathogenic compared to the G2b cluster. The aim of this study was to compare the ability of G1b-based live virus exposure against use of a commercial G2b–based inactivated vaccine to protect growing pigs against G2b challenge. Thirty-nine PEDV naïve pigs were randomly divided into five groups: EXP-IM-1b (intramuscular G1b exposure; G2b challenge), EXP-ORAL-1b (oral G1b exposure; G2b challenge), VAC-IM-2b (intramuscular commercial inactivated G2b vaccination; G2b challenge), POS-CONTROL (sham-vaccination; G2b challenge) and NEG-CONTROL (sham-vaccination; sham-challenge). Pigs were vaccinated/exposed at 3 weeks of age (day post-vaccination 0, dpv 0), VAC-IM-2b pigs were revaccinated at dpv 14, and the pigs were challenged at dpv 28. Among all groups, VAC-IM-2b pigs had significantly higher anti-PEDV IgG levels on dpv 21 and 28 while EXP-ORAL-1b pigs had significantly higher anti-PEDV IgA levels on dpv 14, 21, 28 and 35. EXP-ORAL-1b also had detectable IgA in feces. Intramuscular PEDV exposure did not result in a detectable antibody response in EXP-IM-1b pigs. The fecal PEDV RNA levels in VAC-IM-2b pigs were significantly lower 5–7 days after challenge compared to the POS-CONTROL group. Under the study conditions a commercial inactivated G2b-based vaccine protected pigs against G2b challenge, as evidenced by reduction of PEDV RNA in feces for 3–4 logs during peak shedding and a shorter viral shedding duration. The oral, but not the intramuscular, experimental G1b-based live virus exposure induced a high anti-PEDV IgA response prior to challenge, which apparently did not impact PEDV shedding compared to POS-CONTROL pigs.

## Introduction

Clinical porcine epidemic diarrhea and its causative virus PEDV were discovered in European pigs in the 1970s [1, 2], spread to Asia during the 1980s and 1990s [3], and became endemic in pigs on both continents [2, 3]. Approximately 10 years ago PEDV re-emerged as an important enteric disease of suckling and growing pigs [4]. In 2013, PEDV was introduced for the first time to North America [5] causing major disease and mortality [6].

PEDV can be differentiated into genogroups [7]. On the basis of Spike (S) gene sequences, PEDV isolates can be divided into G1a, G1b, G2a and G2b [7, 8]. G1a includes historic PEDV isolates such as CV777 and attenuated variants distributed in Europe and Asia, whilst G1b includes the so called S-INDEL strains which can be found in Europe, Asia and North America. G2a isolates are restricted to Asia whereas G2b isolates are present in Asia and the Ukraine [9], and since US introduction in 2013 are widespread in the US and considered the US prototype [8, 10]. Differences in pathogenicity between representative isolates of different genogroups have been demonstrated [10–12], with G2b isolates usually being more pathogenic compared to G1b isolates. Partial cross-protection between PEDV G1b and G2b isolates has been demonstrated experimentally [12].

In January 2014 the first conditional licensed PEDV vaccine was introduced to the North American pig market [13], and today an RNA particle-based vaccine and an inactivated PEDV vaccine are available in the US to immunize sows against PEDV [13]. While the use of these vaccines is often beneficial in previously exposed herds, they often fail in naïve herds [14]. One reason for the variable vaccine efficacy observed under field conditions may be the usage of inactivated vaccines given intramuscularly rather than live virus vaccines given orally to induce a strong local enteric immunity. It would be risky to use a known pathogenic G2b live vaccine virus in a pig population; however, using a less virulent variant such as G1b instead may be safe and efficacious.

The objectives of this study were to compare the efficacy of heterologous G1b and homologous G2b based vaccines in protecting growing pigs against G2b challenge. Specifically, an experimental G1b-based live vaccine, administered orally or intramuscularly and a commercial G2b–based inactivated vaccine administered intramuscularly were compared side by side.

## Materials and methods

### Ethical statement

The experimental protocol was approved by the Iowa State University Institutional Animal Care and Use Committee (Approval Number: 5-14-7804-S).

### Animals, housing, and experimental design

**Figure 1:**
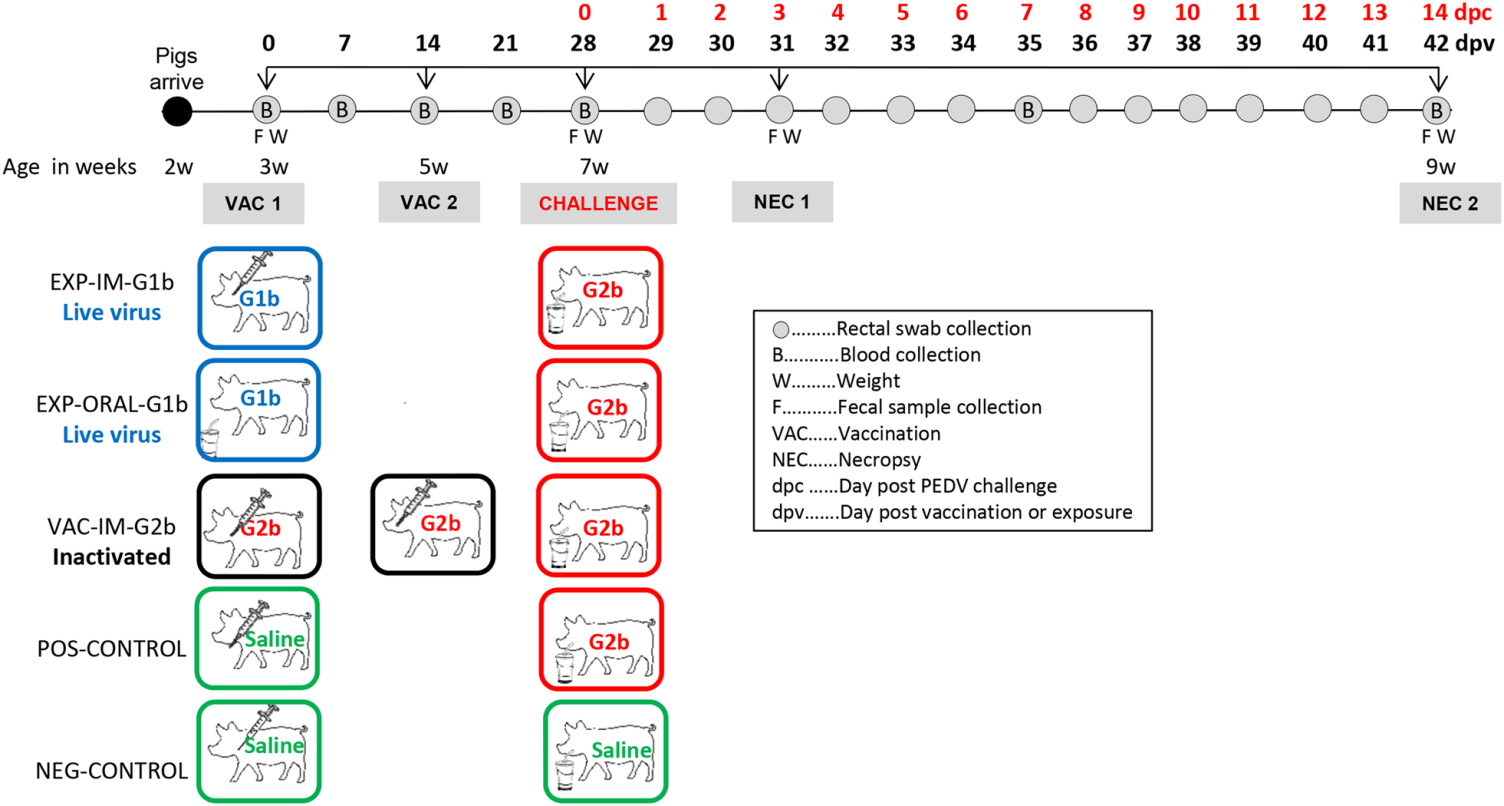
**Experimental design and sample collection.** All abbreviations used in Figure 1 are summarized in the black rectangular box. The top line indicates the day post PEDV challenge and the corresponding day post vaccination at which the main events including vaccinations, G2b PEDV challenge, and necropsies occurred. The pigs were housed in groups of 7–8 and were vaccinated with a live G1b PEDV virus (blue boxes), an inactivated G2b PEDV virus (black boxes) or were sham-vaccinated or sham-challenged (green boxes). Red boxes indicate G2b PEDV challenge.

Thirty-nine, 2-week-old, colostrum-fed, arbitrarily-selected, crossbred, PEDV naïve weaned pigs were randomly assigned to one of five rooms and groups, with 7–8 pigs in each group (Table 1). All groups were fed ad libitum with a balanced, age-appropriate, pelleted feed ration. At 3 weeks of age or dpv 0, EXP-IM-1b, EXP-ORAL-1b and VAC-IM-2b groups were vaccinated with different vaccines and routes as outlined in Table 1, whereas POS-CONTROL and NEG-CONTROL pigs were sham-vaccinated with saline. VAC-IM-2b pigs were revaccinated at 5 weeks of age (dpv 14). At day post-challenge (dpc) 0 or dpv 28, when the pigs were 7 weeks old, they were challenged as shown in Table 1. The POS-CONTROL group served as a challenge control group while the NEG-CONTROL was sham-challenged and served as unvaccinated, unchallenged group. Half of the pigs in each group were necropsied at dpv 31/dpc 3 and the remainder at dpv 42/dpc 14. The experimental design and sample collection details are summarized in Figure 1. Blood was collected in serum separator tubes on a weekly basis (Fisher Scientific, Pittsburgh, PA, USA), centrifuged at 3000 × *g* for 10 min at 4 °C, and the serum was stored at −80 °C until testing. Rectal swabs were collected at dpv 0, 7, 14, 21 and 28 followed by daily collection until dpv 41/dpc 13 using polyester swabs and stored in 5 mL plastic tubes containing 1 mL of sterile saline solution at −80 °C until testing. Individual fecal samples were collected in 50 mL plastic tubes and frozen immediately at −80 °C until testing.

**Table 1:** **Experimental groups, treatments at different days post PEDV vaccination (dpv), average daily gain (ADG) in grams from dpv 0 to 42 (corresponds to day post challenge 14), length and area under the curve (AUC) of PEDV RNA shedding in feces**

Group designation	Number of pigs	Exposure or vaccination					Challenge	ADG^d^	Viral shedding in feces^d^	
		Type	Adjuvant	Genogroup	Route	Timing	dpv 28	dpv 0–42	Length (days)	AUC
EXP-IM-1b	7	Experimental live virus	Adjuplex™	G1b	Intramuscularly	dpv 0	PEDV G2b	412.8 ± 29.8^A^	10.7 ± 1.5^A,e^	236.6^A^
EXP-ORAL-1b	8	Experimental live virus	None	G1b	Orally	dpv 0	PEDV G2b	385.2 ± 27.0^A^	5.8 ± 0.3^A,B^	141.9^A^
VAC-IM-2b	8	Commercial inactivated^c^	Amphigen^®^	G2b	Intramuscularly	dpv 0 and 14	PEDV G2b	402.8 ± 26.1^A^	2.5 ± 1.6^B,C^	43.9^B^
POS-CONTROL^a^	8	Saline	None	NA	Intramuscularly	dpv 0	PEDV G2b	379.8 ± 19.5^A^	7.5 ± 1.3^A^	169.0^A^
NEG-CONTROL^b^	8	Saline	None	NA	Orally	dpv 0	Saline	460.0 ± 22.1^A^	0.0 ± 0.0^C^	0.0^B^

^a^The POS-CONTROL group was sham-vaccinated intramuscularly and inoculated with PEDV G2b and served as an unvaccinated, PEDV challenged group.

^b^The NEG-CONTROL group was sham-vaccinated orally with saline and sham-inoculated with saline and served as unvaccinated and unchallenged control group.

^c^Porcine Epidemic Diarrhea Vaccine; Zoetis.

^d^Data presented as group mean ± SEM.

^e^Different superscripts^(A,B,C)^ within a column indicate significant (*P* < 0.05) different group means.

### Vaccination

At 3 weeks of age (dpv 0), the EXP-IM-1b and the EXP-ORAL-1b pigs were vaccinated with a G1b (US S-INDEL-variant) live PEDV isolate 14-20697 at the 7^th^ cell culture passage [10, 15] as indicated in Table 1. After cell culture adaption this virus was used to infect 5-day old pigs previously and had reduced pathogenicity compared to G2b isolates [10]. For the intramuscular vaccination, 2 mL of the G1b virus stock with a titer of 5 × 10^4^ 50% tissue culture infectious dose (TCID_50_) per mL was mixed with 0.4 mL Adjuplex™ vaccine adjuvant (Lot Number SLBP5255 V; Sigma-Aldrich, St Louis, MO, USA) prior to injection. The same G1b cell culture adapted virus stock used in this study has been shown to have moderate to severe enteric pathogenicity in 5-day old pigs [10]. Each pig in the EXP-IM-1b group received 2.4 mL intramuscularly into the neck, with a total PEDV dose of 1 × 10^5^ TCID_50_. For the oral vaccination route, each EXP-ORAL-1b pig was administered 10 mL of the G1b virus stock with a titer of 6.8 × 10^3^ TCID_50_ per mL by slowly dripping the vaccine into the mouth of each pig with a total dose of 6.8 × 10^4^ TCID_50_. Adjuvant was not used for the oral vaccination route. Pigs in the VAC-IM-2b group were vaccinated intramuscularly with 2 mL of a commercial conditionally-licensed inactivated PEDV vaccine based on a G2b strain (Zoetis; Serial Number 117962) into the right neck. The VAC-IM-2b group was revaccinated 2 weeks later (dpv 14) with another 2 mL of the vaccine as recommended by the manufacturer. The POS-CONTROL group was sham-vaccinated intramuscularly in the neck with 2.4 mL saline and the NEG-CONTROL group was sham-vaccinated orally with 10 mL saline (Table 1).

### Challenge

The 8^th^ passage of virulent PEDV G2b strain 13-19338E [10, 16] was grown to a final titer of 6.8 × 10^4^ TCID_50_ per mL. At 7 weeks of age, EXP-IM-1b, EXP-ORAL-1b, VAC-IM-2b and POS-CONTROL pigs (Table 1) received 10 mL of the PEDV G2b challenge virus stock orally by slowly dripping the inoculum into the mouth with a total dose of 6.8 × 10^5^ TCID_50_. Pigs in the NEG-CONTROL group were sham-inoculated with 10 mL saline orally.

### Average daily weight gain and clinical observations

All pigs were weighed at dpv 0, at dpv 28/dpc 0 and at dpv 42/dpc 14 (Figure 1). The average daily gain (ADG) from dpv 0 (vaccination 1) to dpv 42/dpc 14 (necropsy 2) was calculated. After PEDV challenge the fecal consistency was scored for each pig daily, ranging from 0 to 3 with 0 = solid, 1 = semisolid, 2 = pasty, and 3 = liquid. All pigs were examined daily for other signs of illness including lethargy, respiratory disease, inappetence and lameness.

### Serology

All serum samples were tested for the presence of PEDV IgG and IgA antibodies by an *in*-*house* PEDV G2b S1 protein based indirect ELISA [17, 18]. For IgG detection, a sample-to-positive (S/P) ratio of > 0.2 was considered positive, between 0.14 and 0.2 as suspect, and < 0.14 as negative. For the IgA ELISA an S/P ratio above or equal to 0.14 was considered positive. In addition, fecal samples collected at dpv 0, dpv 28, and at necropsy at dpv 31/dpc 3 or dpv 42/dpc 14 were also tested for presence of PEDV IgA antibodies [18]. Modifications for this assay included that samples were diluted 1:2 and the secondary antibody was diluted 1:2000. The positive cutoff for this assay was S/P ratio equal or greater than 0.14. Serum samples at dpv 28 were titrated for anti-PEDV virus neutralizing antibodies by an immunofluorescence assay as previously described [15]. Serum was diluted two-fold starting from 1:20 to 1:1280. Titers were given as the reciprocal of the last dilution giving a positive result.

### RNA extraction, detection and quantification of PEDV RNA

Total nucleic acids were extracted from all rectal swabs using the MagMax™ Pathogen RNA Kit (Applied Biosystems, Life Technologies, Carlsbad, CA, USA) on an automated nucleic acid extraction system (Thermo Scientific Kingfisher^®^ Flex, Thermo Fisher Scientific, Pittsburgh, PA, USA) according to the instructions of the manufacturer. All RNA extracts were tested for the presence of PEDV RNA by a quantitative real-time PCR [19]. Samples were considered negative when no signal was observed within 40 amplification cycles.

### Necropsy

Half of the pigs in each group were necropsied at dpv 31/dpc 3 and the remaining pigs were necropsied at dpv 42/dpc 14. The pigs were humanely euthanized by intravenous pentobarbital sodium overdose (Fatal Plus^®^, Vortech Pharmaceuticals, LTD, Dearborn, MI, USA). Gross lesions were assessed by a veterinary pathologist and eight sections of small intestines, three sections of large intestines and one section of mesenteric lymph node were collected, fixed in 10% neutral-buffered formalin, and routinely processed for histological examination.

### Histopathology and immunohistochemistry

Microscopic lesions were evaluated by a veterinary pathologist blinded to the treatment groups. Sections of small intestines were evaluated for the presence of villus atrophy and scored from 0 (none) to 3 (severe). PEDV-specific antigen was detected by immunohistochemistry (IHC) using a monoclonal antibody specific for PEDV (BioNote, Hwaseong-si, Gyeonggi-do, Korea) [5, 20]. The amount of PEDV antigen was scored by a pathologist blinded to treatment status. Scores ranged from 0 to 3 with 0 = no signal, 1 = 1–10% of villous enterocytes within the section showing a positive signal, 2 = 11–50% of villous enterocytes showing a positive signal, and 3 = more than 50% of villous enterocytes showing a positive signal.

### Statistical analysis

For data analysis, JMP^®^ software version 11.0.0 (SAS Institute, Cary, NC, USA) was used. Summary statistics were calculated for all the groups to assess the overall quality of the data set including normality. Statistical analysis of the data was performed by one-way analysis of variance (ANOVA) for continuous data. A *P* value of less than 0.05 was set as the statistically significant level. Pairwise test using Tukey’s adjustment was subsequently performed to determine significant group differences. Real-time PCR results (copies per mL of fecal swab suspension) were log_10_ transformed prior to statistical analysis. The area under the curve (AUC) of viral shedding of each animal and the total AUC for each group was calculated using the log transformed values of the viral loads from dpv 29 to 41/dpc 1 to 13. One-way ANOVA and a Bonferroni post hoc test were used to compare groups. Non-repeated nominal data were assessed using a non-parametric Kruskal–Wallis one-way ANOVA, and if significant, pairwise Wilcoxon tests were used to evaluate differences among groups.

## Results

### Clinical observation and average daily weight gain (ADG)

Clinical signs in the PEDV-infected pigs were limited to diarrhea. Three days after vaccination, 4/8 EXP-ORAL-1b pigs had semisolid feces and 7 days later all pigs in this group had pasty feces. None of the pigs in the other groups had any fecal consistency changes and all pigs remained normal until PEDV challenge. Liquid fecal consistency was observed in 4/7 EXP-IM-1b pigs by dpv 31/dpc 3 and feces remained fluid in the majority of the pigs until dpv 35/dpc 7 before becoming pasty-to-solid. In the remaining groups individual pigs had liquid feces for 1–2 days of duration (data now shown) with no differences among groups. The overall ADG is summarized in Table 1. There were no significant differences among groups.

### Anti-PEDV IgG and neutralizing antibody levels in serum samples

**Figure 2:**
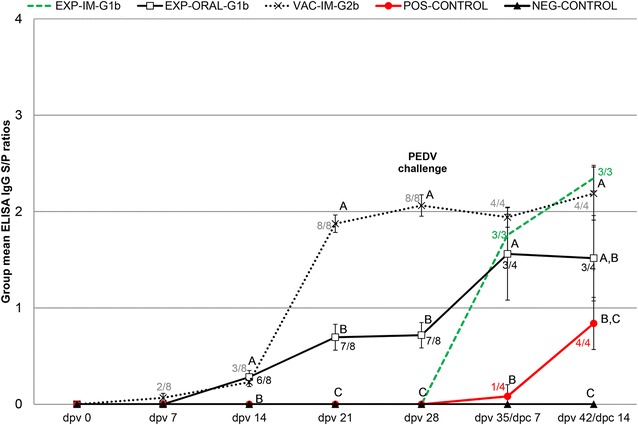
**Group mean anti-PEDV IgG response in serum samples.** The samples were collected at the day of initial vaccination (dpv 0), and dpv 7, 14, 21, 28, 35 and 42 and tested by an *in house* IgG PEDV ELISA. Pigs were exposed to live G1b PEDV at 3 weeks of age (dpv 0) or were vaccinated at 3 (dpv 0) and 5 (dpv 14) weeks of age with a commercial inactivated PEDV G2b vaccine. Pigs were challenged with PEDV G2b at 7 weeks of age (dpv 28/day post challenge or dpc 0). Data presented as mean group ELISA sample-to-positive (S/P) ratios ± SEM. Significantly different values for a dpc are indicated by different superscripts (^A,B,C^). The significance level was set to *P* < 0.05. Seropositive pigs/total number of pigs per group for groups that contained at least one seropositive pigs can be seen next to the group mean.

All pigs were negative for anti-PEDV IgG antibodies in serum samples by ELISA at dpv 0 and NEG-CONTROL pigs remained seronegative for the duration of the study. Anti-PEDV IgG antibodies were first detected in 2/8 VAC-IM-PEDV pigs at dpv 7 (Figure 2). By dpv 14, 3/8 pigs in this group were seropositive and 3/8 were suspect. All VAC-IM-2b pigs were anti-PEDV IgG positive by dpv 21. In the EXP-ORAL-1b group, 6/8 pigs were positive for anti-PEDV IgG antibodies by dpv 14, 7/8 were positive at dpv 21 and at dpv 28, 3/4 pigs were positive by dpv 35/dpc 7, and at termination of the study 3/4 were positive and 1/4 was suspect (Figure 2). The EXP-IM-1b group remained anti-PEDV IgG negative until dpv 35/dpc 7 at which time all three remaining pigs in this group were positive (Figure 2). One of four POS-CONTROL pigs seroconverted to PEDV by dpc 7 and all 4 pigs in this group were seropositive by dpc 14. Anti-PEDV neutralizing antibodies were not detected in the EXP-IM-1b, POS-CONTROL and NEG-CONTROL groups at dpv 28. Neutralizing antibody titers ranged from 40 to 320 with a geometric mean of 89.7 in the EXP-ORAL-1b group, and ranged 40–1280 with a geometric mean of 320 in the VAC-IM-2b group.

### Anti-PEDV IgA antibody levels in serum and fecal samples

**Figure 3:**
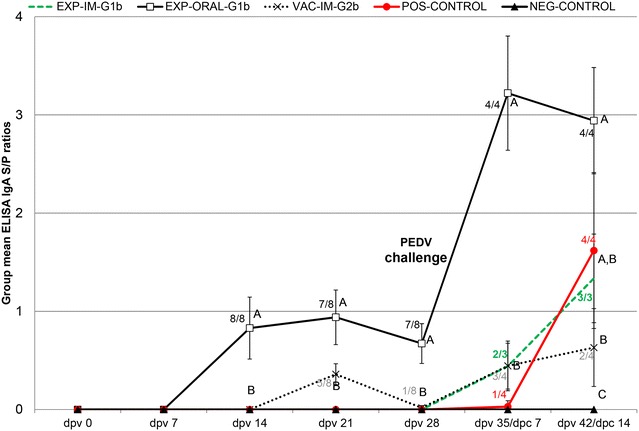
**Group mean anti-PEDV IgA response in serum samples.** The samples were collected at the day of initial vaccination (dpv 0), and dpv 7, 14, 21, 28, 35 and 42 and tested by an *in house* IgA PEDV ELISA. Pigs were exposed to live G1b PEDV at 3 weeks of age (dpv 0) or were vaccinated at 3 (dpv 0) and 5 (dpv 14) weeks of age with a commercial inactivated PEDV G2b vaccine. Pigs were challenged with PEDV G2b at 7 weeks of age (dpv 28/day post challenge or dpc 0). Data presented as mean group ELISA sample-to-positive (S/P) ratios ± SEM. Significantly different values for a dpc are indicated by different superscripts (^A,B,C^). The significance level was set to *P* < 0.05. Seropositive pigs/total number of pigs per group for groups that contained at least one seropositive pigs can be seen next to the group mean.

All pigs were negative for anti-PEDV IgA antibodies in serum samples by ELISA at dpv 0 and 7 and NEG-CONTROL pigs remained seronegative for the duration of the study. Anti-PEDV IgA antibodies in serum samples were first detected in 8/8 EXP-ORAL-1b pigs at dpv 14 (Figure 3). One week later at dpv 21, anti-PEDV IgA in sera were also detected in 5/8 VAC-IM-2b pigs; however, in this group antibody levels decreased, and by dpv 28/dpc 0 only 1/8 pigs were anti-PEDV IgA positive. IgA antibodies against PEDV in the EXP-IM-1b group were detected by dpv 35/dpc 7 in 2/3 pigs and by dpv 42/dpc 14 in 3/3 pigs (Figure 3). One of four POS-CONTROL pigs had detectable anti-PEDV IgA antibodies by dpc 7 and 4/4 pigs were seropositive by dpc 14 (Figure 3). By dpv 28/dpc 0, one EXP-ORAL-1b pig had detectable IgA levels in feces and by dpv 31/dpc 3 one additional pig in this group was positive for PEDV IgA in feces (data not shown). By 14 dpc, PEDV IgA antibodies in feces were present in 2/3 EXP-IM-1b pigs, 3/4 EXP-ORAL-1b pigs, 2/4 POS-CONTROL pigs and 1/4 VAC-IM-2b pigs (data not shown).

### Prevalence and amount of PEDV RNA in rectal swabs and serum samples

**Figure 4:**
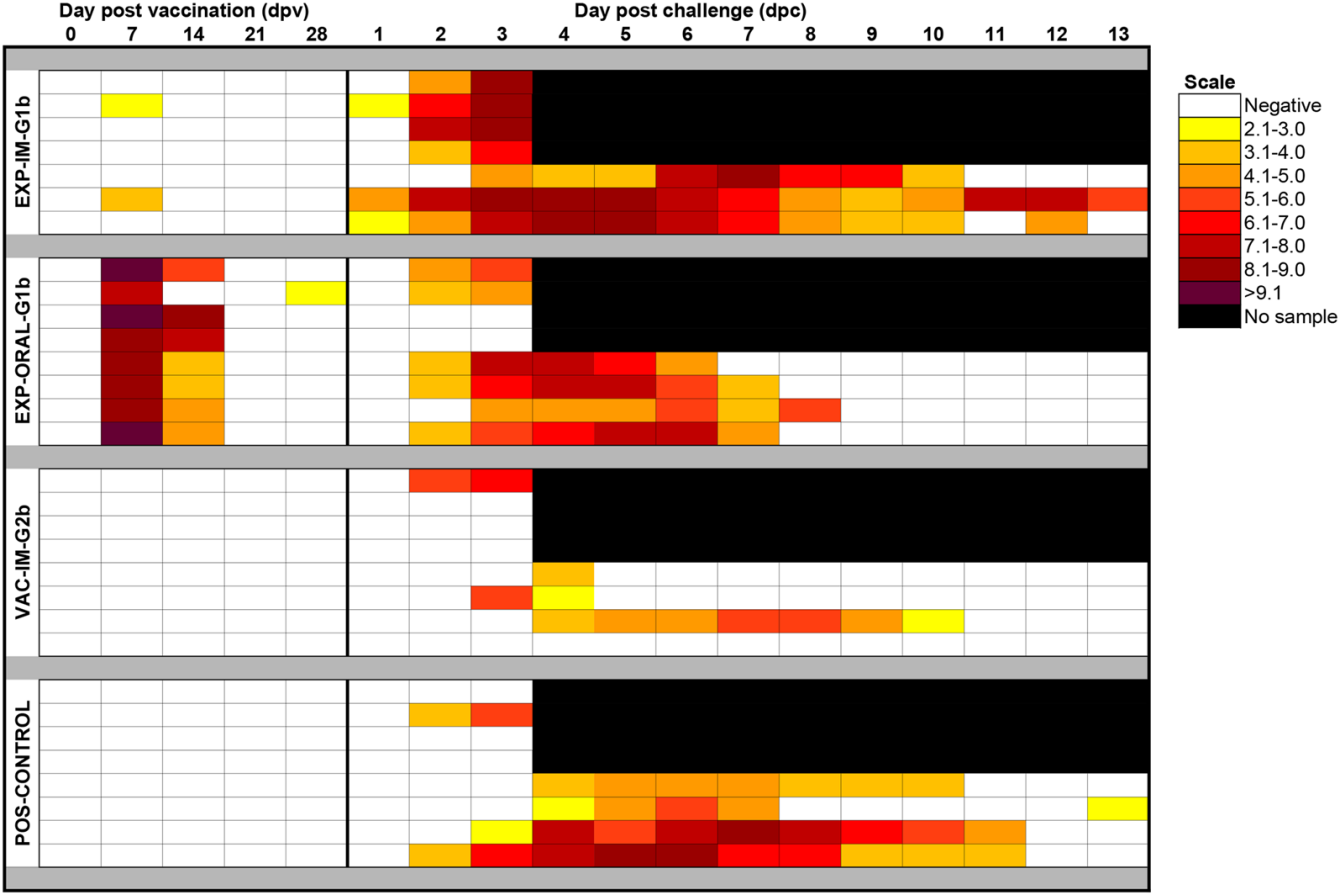
**PEDV shedding patterns for all PEDV challenged pigs separated by group over time.** For each group a line corresponds to the results for a single pig over time and the quantity of virus shed in fecal swabs for all days tested is shown according to the scale on the right side of the heat map. The line between day post vaccination (dpv) 28 and day post challenge (dpc) 1 indicates the PEDV G2 challenge with vaccine virus on the left side and first possible appearance of challenge virus on the right side.

**Figure 5:**
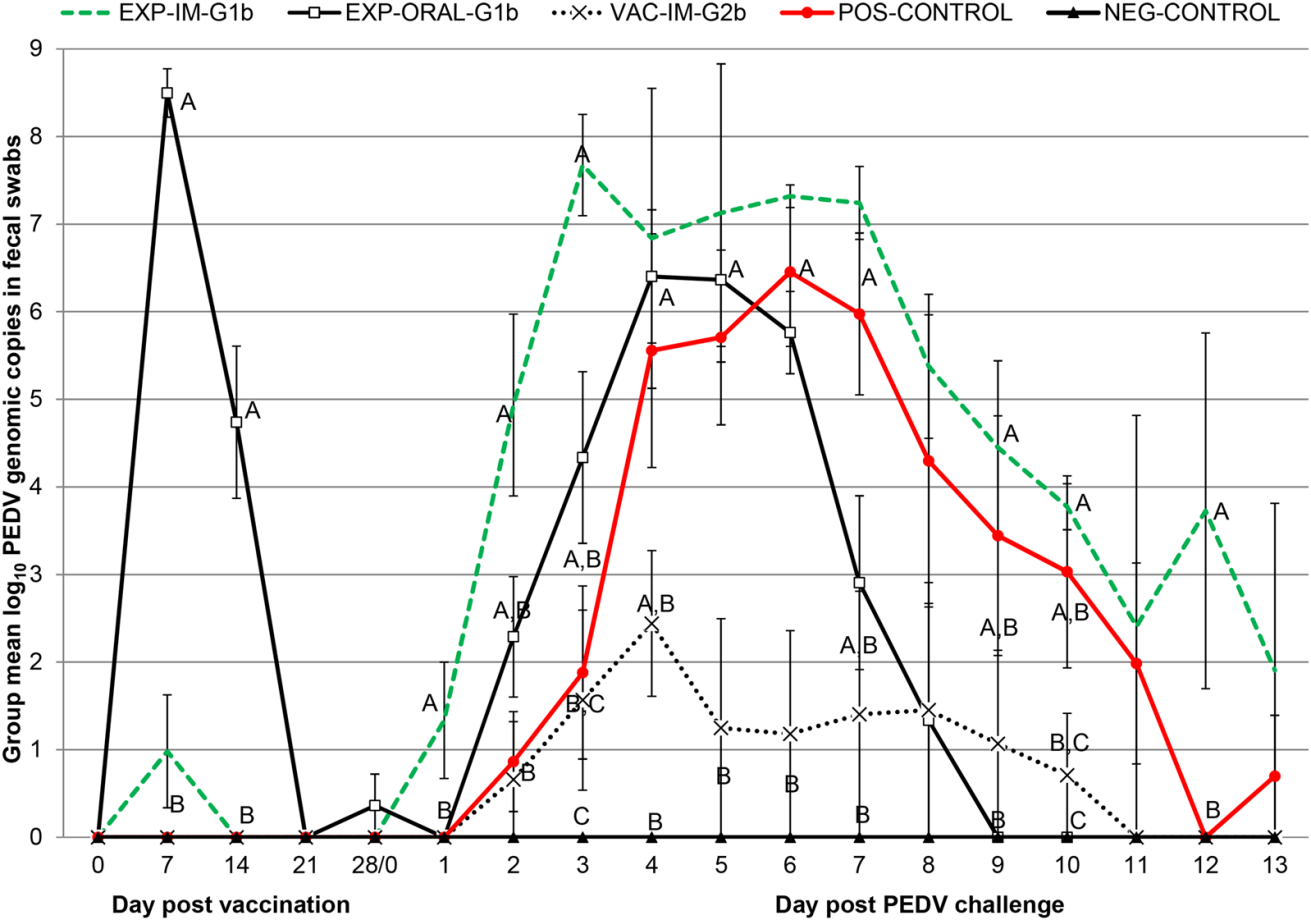
**PEDV RNA shedding in feces at different days post PEDV G2b challenge (dpc).** Pigs were exposed to PEDV at 3 weeks of age (dpv 0) with live PEDV G1b or vaccinated at 3 (dpv 0) and 5 (dpv 14) weeks of age with a commercial inactivated PEDV G2b vaccine. Pigs were challenged with PEDV G2b at 7 weeks of age (dpv 28/dpc 0). **A.** Mean group log_10_ PEDV genomic copies per mL of fecal swab suspension ± SEM. Significant different values for a sample type and dpc are indicated by different superscripts (^A,B,C^). The significance level was set to *P* < 0.05. There were no significant differences among groups on dpv 0 and 21 and on dpc 0, 8, 11 and 13

All pigs were negative for PEDV RNA in fecal swabs on dpv 0 and NEG-CONTROL pigs remained negative for the duration of the study. After vaccination with a live G1b strain, fecal shedding was detected in 8/8 EXP-ORAL-1b pigs by dpv 7 and in 7/8 pigs by dpv 14 (Figure 4). In addition, 5/8 EXP-ORAL-1b pigs had detectable amounts of PEDV RNA in serum by dpv 7 (data not shown). On the day of challenge, 1/8 EXP-ORAL-1b pigs shed low amounts of PEDV in feces. PEDV RNA was detected in rectal swabs of 2/8 EXP-IM-1b pigs by dpv 7; however, PEDV RNA was never detected in serum (data not shown). After challenge, 3/7 EXP-IM-1b pigs shed virus by dpv 29/dpc 1. Viral shedding in rectal swabs was first detected by dpv 30/dpc 2 in 5/8 EXP-ORAL-1b pigs, in 1/8 VAC-IM-2b pigs and in 2/8 POS-CONTROL pigs. Group mean genomic copies of PEDV RNA in rectal swabs are summarized in Figure 5. The average duration of PEDV shedding was calculated by adding the number of consecutive PEDV PCR positive days of each pig that remained in the study until dpv 42/dpc 14 divided by all pigs in a group. The average duration of PEDV shedding and the AUC are summarized in Table 1.

### Gross lesions

At dpv 31/dpc 3, PEDV-infected pigs regardless of vaccination status had hyperemic intestines that were fluid-filled. Specifically, liquid intestinal content was noted in 3/4 EXP-IM-1b pigs, in 2/4 EXP-ORAL-1b pigs, in 2/4 VAC-IM-2b pigs and in 1/4 POS-CONTROL pigs. At dpv 42/dpc 14, 1/4 POS-CONTROL pigs had fluid filled intestines and a dilated colon without remarkable lesions in any of the other pigs.

### Microscopic lesions and PEDV antigen in tissues

Microscopic lesions were seen in 2/4 EXP-IM-1b pigs, 1/4 EXP-ORAL-1b pigs, 1/4 VAC-IM-2b pigs and 2/4 POS-CONTROL pigs which had mild to severe atrophic enteritis by dpv 31/dpc 3. There were no lesions in any of the other pigs. Five of the six pigs with microscopic lesions also had moderate-to-high amounts of PEDV antigen associated with the lesions (two EXP-IM-G1b pigs, scores 3 and 3; a EXP-ORAL-G1b pig, score 2; a VAC-IM-G2b pig score 3; and a POS-CONTROL pig, score 3). There were no significant differences in antigen levels or severity of microscopic lesions among groups. No microscopic lesions nor PEDV antigen were observed at dpv 42/dpc 14.

## Discussion

Vaccination strategies to protect against PEDV are challenging, as the most vulnerable population is suckling pigs. Vaccine efficacy studies using pregnant sows are difficult and costly. To select novel PEDV vaccine candidates and to generate preliminary data, the growing pig model has been used [21]. In this study growing pigs were used to test and compare the efficacy of live or inactivated vaccines to protect pigs against challenge with a highly virulent G2b PEDV isolate.

Pig veterinarians and producers often prefer intramuscular administration to assure each pig gets vaccinated with the appropriate dose. Intramuscular administration is known to induce a systemic immune response [22]. In this study, VAC-IM-2b pigs had a strong anti-PEDV IgG response in serum which was significantly higher compared to EXP-ORAL-1b pigs. This could be due to the adjuvant Amphigen^®^ used in the commercial product or due to the booster dose that the VAC-IM-2b pigs received. In contrast to live virus exposure, inactivated vaccines are almost always given in 2 dose regimens; hence in this study the VAC-IM-G2b group received a booster dose whereas the EXP-IM-G1b and EXP-ORAL-G1b pigs did not. In contrast to oral exposure to a live virus, pigs vaccinated with the commercial inactivated virus had a weak anti-IgA response in serum and no anti-PEDV IgA response in feces. This is not surprising as inactivated vaccines often do not induce effective mucosal immunity in naïve pigs whereas oral exposure elicits better gut immunity [22]. It has been shown that IgA levels in serum correlates with IgA measured in feces from experimentally infected piglets [18] and in serum and colostrum and milk samples of sows orally immunized [13]. These studies indicate that measuring IgA levels in serum samples may be a marker of protection.

For safety reasons, veterinarians and producers often prefer inactivated vaccines. However, for some viruses such as porcine reproductive and respiratory syndrome virus (PRRSV), it has been shown that inactivated vaccines are largely ineffective [23]. PRRSV requires live virus to migrate to the lung and replicate at low levels to induce protection. Similarly, PEDV may also require local activation of the gut-associated mucosal system. In Asia, where PEDV vaccines have been available for decades, attenuated G1a-based intramuscular vaccines are commonly used [4, 24]. We attempted to inject a G1b isolate intramuscularly with an adjuvant. Under the study conditions, except for 2/7 EXP-IM-1b pigs with low levels of PEDV RNA in rectal swabs at 7 dpv, there was no sign of infection in this group based on lack of seroconversion and lack of detectable PEDV RNA in serum or feces. The two PEDV RNA positive EXP-IM-1b samples were retested and results confirmed (data not shown).

The pigs that were vaccinated intramuscularly with a commercial G2b vaccine were protected against homologous G2b challenge as evidenced by reduction of the amount of PEDV RNA in feces by 3–4 logs during peak shedding between dpc 5–7 (dpv 33–35) and shortening of the duration of viral shedding. Viral titers to determine infectivity were not determined, but it has been shown previously that contact pigs can be infected for up to 14 days after initial infection of a seeder pig group [25]. In this study a homologous G1b challenge for pigs vaccinated with the experimental G1b live vaccine was not included due to space and cost reasons. In addition, G2b isolates, considered to be more pathogenic compared to G1b isolates [10, 12], appear to be the primary cause of clinical disease associated with PEDV under field conditions and are more widely distributed compared to G1b isolates. Pigs orally vaccinated with an experimental heterologous G1b live vaccine had a tendency for a shortened viral shedding duration; whereas pigs vaccinated intramuscularly with an experimental heterologous G1b live vaccine were not protected. It has been shown that piglets orally inoculated with a virulent CV777 strain were fully protected after challenge, while protection was not complete in pigs orally inoculated with an attenuated CV777 strain [26]. Prior to usage the G1b stock was passaged seven times which could have resulted in a low degree of attenuation. It is worth noting that pigs orally immunized with the G1b live vaccine presented mild diarrhea and shed high levels of virus for at least 2 weeks after immunization. This could pose risks of infection and potentially more serious clinical signs in younger piglets.

Results from studies on cross-protection between genogroups have been contradictory. A previous study showed that although G1a-based vaccines (CV777 and DR13 strains) could provide protection against homologous challenge, they were not protective against contemporary Chinese G2b strain YC2014 [27]. It has been suggested that sows naturally-infected with a G1b strain produce heterologous lactogenic protective immunity against G2b strains 7 months after initial infection [28]. However, infection of 3–4 day old piglets with G1b strain provided variable protection against a G2b challenge 21–29 days later and the extent of protection was shown to be litter-dependent (mortality 0 to 75%) [12]. Additionally, the antigen concentration in the commercial (10^6^–10^8^ TCID_50_/dose) and experimental (10^4^–10^5^ TCID_50_/dose) intramuscular vaccines may have contributed to differences in the protection observed in the current study. The dose of experimental vaccine was limited by the G1b virus titer achieved after propagation.

Under the conditions of this study, a commercial inactivated G2b-based PEDV vaccine administered intramuscularly protected pigs against homologous challenge. In contrast, an experimental G1b-based live virus vaccine given intramuscularly was not protective. The same virus given orally induced a high IgA response but the virus shedding pattern after challenge mimicked that of the POS-CONTROL group suggesting limited protection. This could perhaps indicate that induction of a genotype specific humoral and/or cellular immune response may be important for PEDV protection.
